# Statins and Male Fertility: Is There a Cause for Concern?

**DOI:** 10.3390/toxics10100627

**Published:** 2022-10-20

**Authors:** Temidayo S. Omolaoye, Mouhammad Omar Halabi, Maitha Mubarak, Asha Caroline Cyril, Ruthwik Duvuru, Rajan Radhakrishnan, Stefan S. Du Plessis

**Affiliations:** 1 College of Medicine, Mohammed Bin Rashid University of Medicine and Health Sciences, Dubai P.O. Box 505055, United Arab Emirates; 2 School of Medicine, Royal College of Surgeons Ireland-Bahrain, Busaiteen 15503, Bahrain; 3 Division of Medical Physiology, Faculty of Medicine and Health Sciences, Stellenbosch University, Tygerberg, Cape Town P.O. Box 7505, South Africa

**Keywords:** statins, male fertility, testicular dysfunction, steroid hormones, simvastatin, rosuvastatin, atorvastatin, fluvastatin, lovastatin, pravastatin

## Abstract

The well-known 3-hydroxyl 3-methyl glutaryl-Coenzyme A reductase inhibitors, called statins, have been the main medication used in the treatment of hypercholesterolemia and some cases of cardiovascular diseases. The effectiveness of this drug in controlling cholesterol production is impeccable, however, patients often complain of a variety of side effects, such as myalgia, muscle atrophy, and in some cases, rhabdomyolysis. Not only has the use of statins caused the aforementioned side effects, but they are also shown to cause testicular discomfort, erectile dysfunction, altered semen parameters, and modified steroid hormone production. These reported adverse effects on male fertility are not generally agreed upon, as some have shown the use to be beneficial. Hence, this makes the aftermath effect of statin use on male fertility debatable and controversial. The negative effects have been associated with imbalanced or reduced steroid hormones, which are necessary for proper spermatogenesis and other sexual functions. Meanwhile, the beneficial effects are related to statin’s anti-inflammatory and cardioprotective properties. These contradictory findings are in part due to the different age of users, concentrations of statins, the type and duration of treatment, and the underlying disease and/or comorbidities. Therefore, the current study aims to analyze the literature and gather evidence as to the effects of statin on male sexual health and reproductive parameters, and subsequently give recommendations for the direction of future studies.

## 1. Introduction

For decades, statins (3-hydroxyl-3-methyl glutaryl-coenzyme A (HMG-CoA) reductase inhibitors) have been a keystone treatment for hypercholesterolemia and are commonly used in clinical practice for the primary and secondary prevention of cardiovascular disease on account of their cholesterol-lowering properties [1,2]. Despite the widespread use of statins due to their effectiveness in controlling cholesterol production, there are alarming concerns about the side effects, such as myalgia, muscle atrophy, and in some cases, rhabdomyolysis. Similarly, statins have remained the subject of question concerning their effect on male reproductive physiology and sperm parameters.

For sperm to fully develop, the testis requires cholesterol for the synthesis of testosterone, which in turn is needed for spermatogonia proliferation and differentiation; and the developed sperm must undergo remodeling of the lipid membrane in the epididymis to facilitate motility. Likewise, cholesterol efflux from the plasma membrane is needed for capacitation [3], and capacitation is necessary for sperm to penetrate the zona pellucida to fertilize the oocyte. Since impaired lipid metabolism promotes infertility [4], statins could potentially have the ability to regulate lipid metabolism through various molecular pathways, and fertility should be improved, as seen in some cases of hypercholesterolemia. Nevertheless, it is not that simple, because testosterone, which is required for the entire process of spermatogenesis, is a steroid hormone and requires cholesterol as a precursor, but statins lower cholesterol levels. Additionally, statins have also been shown to have an inhibitory effect on coenzyme Q10, an enzyme which protects against oxidative stress [5]. This phenomenon has presented a genuine concern about the effect of statins on male reproductive function.

To date, reports from both human and animal studies have remained contradictory, as there is evidence demonstrating both the lack of consequences and adverse effects of statins on male fertility. For example, a population-based epidemiological study performed among 1812 men as a part of the Boston Area Community Health (BACH) survey in the US in 2007 showed that it is less likely for statins to lower serum androgen levels [6]. Similarly, Keihani et al. reported that the semen parameters of men who were on statin therapy for more than three months remained unchanged except for the semen volume, which was reduced [7]. In opposition, a study that investigated the effects of prolonged simvastatin treatment (up to 12 months) on the testicular function of hypercholesterolemic male patients showed a significant decrease of both basal and human chorionic gonadotropin-stimulated free testosterone levels at 6 and 12 months [8]. Similar observations were also reported in other human studies on different forms of statin [7,9,10,11], with the inclusion that statins can worsen erectile dysfunction [3,12]. Studies using rodent models of statins have reported several adverse effects including a decrease in primary spermatocytes, sperm motility, normal morphology, and reduced sperm count [13,14,15,16,17,18,19,20,21].

Although some studies demonstrate that statins may have serious adverse effects on male fertility, several others showed no effect. Therefore, this review aimed to analyze the current literature regarding the effect of statin treatment on male reproductive and sexual health. Additionally, a brief background on statin, its metabolism and mechanism of action is described.

## 2. A Brief Overview of Statins

Statins are HMG-CoA reductase inhibitors that manage the rate of cholesterol production within the body. Statins repress the HMG-CoA reductase enzyme required for mevalonate synthesis, a naturally occurring substance essential for de novo sterol development and cholesterol production. In response to HMG-CoA reductase inhibition, there is reduced production of cholesterol and low-density lipoprotein cholesterol (LDL-C), depending on the statin and dose, as the types of statins vary in clearance efficacies [22].

Seven members of the statin drug class are currently FDA-approved and available for therapeutic administration. This includes atorvastatin, fluvastatin, lovastatin, pitavastatin, pravastatin, rosuvastatin, and simvastatin [23]. Based on their physico–chemical properties, statins are categorized into lipophilic and hydrophilic groups. The lipophilic statins include simvastatin, fluvastatin, pitavastatin, lovastatin and atorvastatin, while hydrophilic statins include pravastatin and rosuvastatin. It is important to note that the target organ for all statins is the liver. However, when there is residual statin and metabolites that were not taken up by the hepatocytes, then the residuals are distributed into various systems through systemic circulation [24]. Studies have shown that the systemic circulation concentration of atorvastatin is 12%, simvastatin is 5%, pravastatin is 17%, fluvastatin ranged between 20–30%, while lovastatin is 5% [24].

In statin therapies, individualized patient care is essential as the drug-specific effects are case-dependent. Thus, pharmacokinetic properties are considered in the approach to appropriate statin selection for cardiovascular disease intervention. Jones et al. described the dose efficacy of different statins following categorization into three groups in accordance with their LDL-C reduction intensity, determined by the individual dose [25]. The findings of the study evidenced a greater reduction of LDL-C using atorvastatin in 10 mg, whereas low LDL-C reduction rates were observed using simvastatin 10 mg, pravastatin 10 mg and 20 mg, and fluvastatin 20 mg and 40 mg [25]. Likewise, Chong et al. examined the available statins to determine possible distinctive clinical properties and advantages. The obtained results demonstrated the statins’ differing ability in reducing LDL-C levels, varied pharmacokinetics, and food-drug interactions [26].

Natural and synthetic statins share efficacy in lipid profile improvement. Natural statins include pravastatin and simvastatin, which are of fungal origin, whereas synthetic statins that include atorvastatin and fluvastatin are obtained from mevalonate and pyridine. Upon exploration, it was found that atorvastatin and simvastatin would be the choice options for patients that failed to achieve the specified LDL target [27].

## 3. Mechanisms of Action

**Figure 1:**
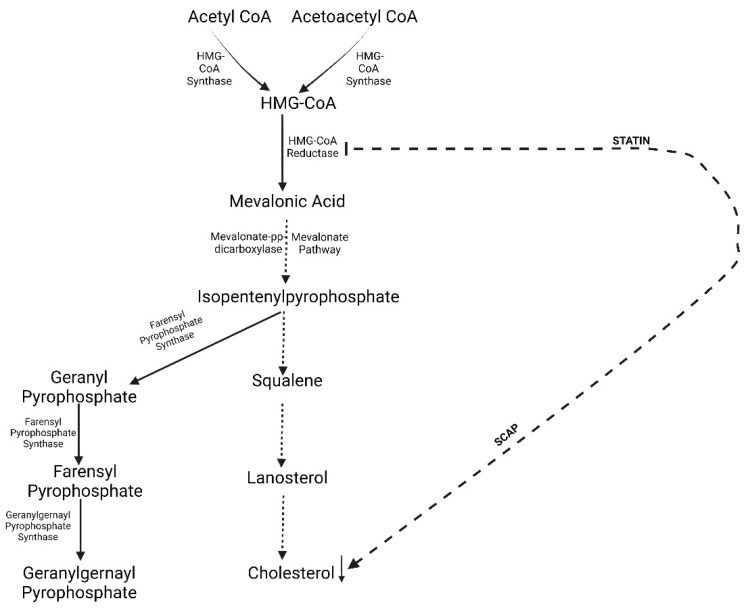
The mechanism of the action of statin. Acetyl CoA or Acetoacetyl CoA are converted to HMG-CoA by HMG-CoA synthase and HMG-CoA is converted to mevalonic acid by HMG-CoA reductase. This latter step is crucial in the formation of cholesterol. To mediate the formation of cholesterol, statins inhibit the enzyme HMG-CoA reductase. HMG-CoA reductase synthesis is regulated by the steroid regulatory element binding proteins (SREBP). The binding of SCAP to SREBP activates HMG-CoA reductase. Therefore, if SCAP is inhibited, then HMG-CoA reductase will be inhibited and will consequently inhibit cholesterol synthesis. Cholesterol is also able to mediate its own availability by either inhibiting or activating SCAP. ↓ = decrease.

Statins share common mechanisms of action but differ in lipid-modifying efficacy, chemical structures, and pharmacokinetic profiles. They have three important parts: an analogue of HMG-CoA (modified 3,5-dihydroxyglutaric acid), which is structurally similar to endogenous HMG-CoA, a side chain ring structure that determines the solubility, and a complex ring structure that enables the binding of the statin to the HMG-CoA reductase enzyme. After statin administration, the active components aid the decrease in plasma LDL-C through the inhibition of the HMG-CoA reductase present in the liver and the cholesterol biosynthetic pathway [28]. Consequently, the process of HMG-CoA to mevalonate conversion is repressed, causing a reduction in cholesterol production. The LDL receptor transportation is also activated, leading to the increase of LDL receptors which amplifies LDL-C uptake and reduces the plasma LDL-C levels [29]. This is briefly summarized in Figure 1.

## 4. Metabolism of Statins and Toxicokinetics

All statins are biotransformed in the liver, thus are metabolized by the cytochrome P450 (CYP450) isoenzyme, aside from pravastatin, which is metabolized in the cellular cytosol by sulfation. The CYP450 isoenzyme is located in the lipid bilayer of the endoplasmic reticulum of hepatocytes, therefore, it is predominant in drug-metabolism interactions, bioactivation, and conducts the microsomal metabolism of statins [30]. Atorvastatin and simvastatin are metabolized in the CYP34A pathway, whereas fluvastatin is metabolized via CYP29. Rosuvastatin is not extensively metabolized, however, is mainly metabolized by CYP2C9, whereas pravastatin does not undergo metabolism by the CYP450 system [31]. Thus, these differences result in varied pharmacokinetic features between each statin. Studies have also shown that the inhibition of HMG-CoA reductase will also reduce the synthesis of ubiquinone, steroids, bile acids, vitamin D, geranylgeranyl pyrophosphate (GGPP), and farnesyl pyrophosphate (FFP) [32]. The latter metabolites (GGPP and FFP) are important for cell growth maintenance, prevent apoptosis, and aid in the reduction of the cholesterol content in skeletal muscle cells.

Unfortunately, discontinuation rates of statin therapy are high despite the general tolerability of the medication. This is in part due to the failure to resume the full course of treatment because of the fear of developing symptoms associated with drug interactions or the adverse effects of statin usage, such as myotoxicity, new-onset diabetes, and other complications [2,27,33,34].

Statin-related myotoxicity (SRM) is a common adverse event of statin usage that occurs within the first year of treatment commonly due to either the *nocebo* and/or actual effect, resulting in varying degrees of exertional fatigue, disorientation, and amnesia. The clinical spectrum of SRM includes maximizing cardiovascular morbidity and mortality. SRM is characterized by generalized muscular pain in addition to muscular weakness, stiffness, and cramps in the thighs and calves attributable to the increase in creatine kinase concentration, myositis, rhabdomyolysis, tendinitis, and myalgia. Symptoms associated with SRM have an estimated onset of six months with resolution reported to occur two months after statin discontinuation. Although the wide spectrum of SRM has received great attention, the pathogenesis of SRM has not been clearly explained. Nevertheless, mechanisms that involve the disruption of the supply of farnesyl and geranyl pyrophosphate, or via mitochondrial dysfunction and calcium ion leakage have been implicated. These are hypotheses, but in addition, another important risk factor is the genetic predisposition to develop SRM, as these symptoms are not always observed in all patients. Other conditions that have also been associated with the use of statins include neurological and cognitive conditions, hepatotoxicity, renal toxicity, cataracts, gastrointestinal effects, urogenital effects, and gynecomastia [35].

Not only have the use of statins caused the aforementioned side effects, but they have also been shown to cause testicular discomfort, erectile dysfunction, altered semen parameters, and modified steroid hormone production. These reported adverse effects on male reproductive parameters are not generally agreed upon, hence, making the aftermath effect of statin use on male fertility debatable and controversial.

The remainder of this review highlights what is currently known about the effect of different forms of statin on male reproductive health and parameters, justifying with findings from both human and animal studies.

## 5. Search Method

A systematic literature search was conducted on the different forms of statin and their plausible impact on male reproductive health and parameters. The literature search did not discriminate against papers published before a certain date. Data were retrieved from diverse credible literature sources and electronic databases including PubMed, PubMed Central, Medline, Google Scholar, and Cochrane.

## 6. Evidence of the Effect of Different Statin Forms on Male Reproductive Parameters

To address what is currently known on the effects of statin on male reproductive parameters and testicular function, this section highlights each known statin and also describes their effects on male fertility (Table 1).

### 6.1. Atorvastatin and Male Infertility

Atorvastatin (ATZ) is one of the first-line drugs against hyperlipidemia, and it can be prescribed at 10, 20, 40, and 80 mg/day [57]. Different doses of atorvastatin exhibit varying effects. For example, a low dose of atorvastatin may have an anti-inflammatory effect and antioxidant properties [58,59], while a high dose of Atorvastatin may negatively alter sperm motility and morphology [47,60].

#### 6.1.1. Animal Findings

Animal studies have investigated the effect of atorvastatin on male fertility by either treating healthy rats with atorvastatin or by inducing a disease state in these animals and thereafter determining the effect of atorvastatin. A study by Akdeniz et al. evaluated the effects of atorvastatin in a cohort of male Sprague–Dawley rats exposed to 20 mg/kg of atorvastatin per day for 12 weeks. Following treatment, findings showed a decrease in the number of Sertoli cells, spermatogonia, and spermatocytes when compared to the control group [36]. Although this dose was low, it still had a negative effect on spermatogenesis. In contrast, a study demonstrated the benefits of atorvastatin as a protective drug against busulfan-induced spermatogenesis injury in Wistar rats. Administration of atorvastatin commenced after the first day of the busulfan therapy and was continued for 28 days. Following treatment, spermatozoa were collected from the epididymis and the testicular tissue was used for all biochemical analyses. The results showed that atorvastatin was protective against oxidative stress-related injuries in rats [37]. Naeimi et al. reported similar findings on a cohort of BALB/c mice; they found that atorvastatin was protective against ionizing radiation (IR). After histological examination, they found a significant difference between testicular injury in the IR group and the atorvastatin group. The IR group showed testicular atrophy with cellular damage, whereas the atorvastatin group showed slight cellular damage, and interstitial tissue was found to be preserved [38].

The contradictory findings seen in these animal studies are plausibly due to variability in disease presentation. The study that reported the adverse effect of atorvastatin was performed on healthy rats while the studies that showed the beneficial effects were carried out on diseased animals. This implies that treatment with atorvastatin may be hazardous for proper testicular function when used under normal conditions.

#### 6.1.2. Human Findings

The study of Pons et al. evaluated the effect of atorvastatin (10 mg) on a cohort of 17 young, healthy males for a five-month period. Blood and semen parameters (morphology and motility) were measured before and during treatment, as well as for three months after treatment. Capacitation was evaluated using two main pathway signaling markers. Cholesterol redistribution in sperm and the phosphorylation of tyrosine residues of terminal protein markers were also assessed. During the treatment period, reduced cholesterol and LDL-C were observed, which returned to the pre-treatment values after withdrawal of atorvastatin. The cholesterol reduction did not affect semen cholesterol and phospholipid levels and sperm motility was observed to have improved. However, total sperm count was noted to have decreased significantly (*p* < 0.05) after treatment, and abnormalities in the head, neck, and midpiece were also significantly increased (*p* < 0.05) [47]. Additionally, atorvastatin negatively affected the capacitation of sperm up to 3 months after treatment; such findings denote the negative effects of atorvastatin on sperm parameters and male fertility [47]. Furthermore, since atorvastatin influenced the sperm capacitation adversely, this could lead to pernicious influences on male fertility, as capacitation is the key for fertilization to occur.

Seeing that both the animal study that investigated the effect of atorvastatin on healthy rats [36], and the human study that examined atorvastatin on young healthy males [47] reported similar findings regarding its negative impact on proper testicular function and reproductive parameters, proper care while prescribing or co-administering atorvastatin is recommended.

### 6.2. Fluvastatin and Male Infertility?

Fluvastatin was the first synthetic statin derived from pyridine, which is effective in reducing blood cholesterol [61], and has been reported to be relatively safe [62]. Currently, there is no clinical trial available regarding the effect of fluvastatin on male fertility and available animal studies have only investigated its potential ameliorative effects in diseased conditions.

#### Animal Findings

Since statins have been thought to be protective against oxidative stress, several animal studies attempted to examine such protective effects. A study by Imaeda et al. examined the anti-oxidative effects of fluvastatin on mammalian derived CHL/IU cells from a cohort of Chinese hamsters. The CHL/IU cells were exposed to hydrogen peroxide or t-butylhydroperoxide and oxidative damage was assessed. At varying concentrations, employing the comet assay, both hydrogen peroxide and t-butylhydroperoxidase caused increased tail length and movement of DNA. Administration of fluvastatin caused a marked reduction in tail movement. These results demonstrate that fluvastatin may have radical scavenging properties, which enables it to have a protective factor against DNA damage and mutagenesis [54].

Another study examined such protective effects in a cohort of obesity-induced Sprague–Dawley rats. Exposure to fluvastatin was done for eight consecutive weeks, after which the rats were euthanized, and examination of the testes was performed. The tests included the testicular weight and gross morphology, sperm parameters, circulating sex hormones, lipid levels, and the tissue mammalian target of rapamycin (mTOR)/Ribosomal protein S6 kinase beta-1 (p70s6k). The researchers found that exposure to fluvastatin prevented testicular atrophy, promoted sperm quality, and increased the number of spermatogonia, Leydig, and Sertoli cells. The hormone levels were shown to be altered by high cholesterol levels, and thus, exposure to fluvastatin helped to restore normal FSH, LH, and testosterone levels. This was attributed to the blood lipid-lowering effect rather than the anti-oxidative properties of fluvastatin. The study suggested that the aforementioned protective qualities of fluvastatin on sperm were due to promoting the expression of mTOR and p70s6k, which are expressed in spermatogonia [40].

These results were further confirmed in a study in 2019 by Gurel et al., which examined the effects of doxorubicin-induced testicular toxicity in a cohort of male Sprague–Dawley rats. They found that by promoting the expression of mTOR, fluvastatin was able to reduce the effects of doxorubicin toxicity [41]. In line with the previous findings on the effects of atorvastatin in animal models, fluvastatin seems to have similar beneficial effects on sperm parameters in diseased conditions, which is owed to its antioxidant properties.

To date, no studies have evaluated the effects of fluvastatin on human male fertility. However, due to the evidence of increased mTOR expression in animal models, fluvastatin has been used for cancer therapy. A study showed that fluvastatin administered with vorinostat for renal cancer had positive effects, showing that mTOR expression was increased in human models [55]. A more recent study in 2021 by Wang et al., evaluated the anti-oxidative effects of fluvastatin on neuronal cells. The researchers exposed neuronal cells to pathologic oxidative stress via H_2_O_2_ and thereafter administered fluvastatin. Upon treatment, fluvastatin was found to decrease the effects of H_2_O_2_ on the neuronal cells in a dose dependent manner [56]. The upregulation of mTOR reduced oxidative stress, by promoting mitochondrial oxidative metabolism [63]. Although no studies have evaluated the effects of fluvastatin on sperm parameters, the aforementioned studies were still able to demonstrate the beneficial antioxidant effects of fluvastatin. Nevertheless, further studies are needed to establish a baseline understanding of the effects of fluvastatin on male reproductive parameters; whether it affects male fertility remains debatable.

### 6.3. Pravastatin and Male Infertility

#### 6.3.1. Animal Findings

Animal model studies have assessed the anti-oxidative effects of pravastatin following the induction of different disease states. A study by Farsani et al. examined such properties on a cohort of 24 healthy male Sprague–Dawley rats that were exposed to doxorubicin. After eight weeks of treatment, further analyses were performed. Histological examination revealed a decreased number of spermatocytes and a reduction in germ cells. While the biochemical analysis showed elevated malondialdehyde (MDA) levels and declined glutathione peroxidase (GPx) and superoxide dismutase (SOD) activities when doxorubicin was administered. These adverse effects were alleviated upon treatment with pravastatin [44]. Such antioxidant effects were also reported by Dursun et al. and Esrafil et al. [45,64].

Another study examined the antioxidant effects of pravastatin in a cohort of hypertensive and normotensive Wistar rats. It was reported that the antioxidant effects of pravastatin could be attributed to inhibiting HMG-CoA reductase, which is the enzyme that catalyzes the formation of mevalonic acid. Through this, pravastatin could inhibit the production of the isoprenoid intermediates that prevent the NADPH-oxidase intermediates [46]; this can limit the stress on the spermatozoa and prevent the further generation of reactive oxidative species (ROS) by the mitochondria, which can limit ROS-induced apoptosis [65] and potentially protect the integrity of the DNA and promote better fertilization and pregnancy outcomes [66].

Based on these findings, conclusions on the effect of pravastatin on reproductive parameters in animal models cannot be reached because the respective studies only investigated induced diseased states that are not related to hypercholesterolemia. However, these findings may be useful for informing about drug co-administration.

#### 6.3.2. Human Findings

Berini et al. evaluated the effects of pravastatin in a cohort of eight hypercholesterolemic males. All patients were given a placebo for four weeks, followed by pravastatin (20 mg) for six months. Throughout the study, they evaluated lipid levels, hormones, testicular function, which was measured by the administration of HCG, and spermatogenesis. Upon treatment, they observed a significant decrease in LDL levels, while semen parameters remained unchanged; suggesting that the long-term therapy of pravastatin does not affect male reproductive function [19]. These results are supported by another study that reported no significant change in sperm parameters after assessing the effect of pravastatin on adrenocortical and testicular steroidogenesis [48].

Additionally, Dobs et al. evaluated the effects of pravastatin on reproductive functions in hypercholesteremic males. The subjects were divided into two groups; one of the groups was given pravastatin (40 mg) and placebo cholestyramine powder, while the other was given placebo pravastatin tablets and 16 mg of cholestyramine powder. Endocrine testing was performed at 6, 12, 18, and 24 months after randomization; cortisol levels, aldosterone, and dihydroepiandrostenedione levels were measured. The gonadal function was measured by evaluating the basal levels of FSH, LH, prolactin, and progesterone. Semen analysis was also performed after the intramuscular injection of HCG 2500 IU. Following treatment, no significant changes were detected in gonadal and endocrine parameters; however, there was a significant reduction in sperm motility (*p* < 0.05), sperm concentration, and a non-significant decrease in morphologically normal sperm [9]. The limitation of the study is that the reproductive parameters were not measured before starting the study.

Since available findings are contradictory, it is precarious to draw a conclusion as to the effects of pravastatin on male reproductive function, especially from a human studies’ perspective. However, from an animal studies standpoint, pravastatin may have some anti-oxidative effect when subjected to an external stressor. Taken together, larger studies with humans are needed to reach any definitive conclusions.

### 6.4. Rosuvastatin and Male Infertility

Rosuvastatin is a synthetic HMG-CoA reductase inhibitor. It has an additional stable polar methane–sulphonamide group which increases its interaction with HMG-CoA reductase and decreases the lipophilicity of the drug, making it unlike any other statin [67]. Rosuvastatin’s low lipophilicity and decreased extrahepatic tissue penetration cause around 90% of the drug to be excreted, allowing for a favorable side effect profile [67]. With rosuvastatin being one of the most effective statins in managing hypercholesterolemia [68], it is essential to evaluate its effects on male fertility.

#### 6.4.1. Animal Findings

In animal models, Leite et al. evaluated the effects of rosuvastatin on male fertility in a cohort of Wistar rats. The study assessed whether rosuvastatin would have an effect on prepubertal males, to measure the safety of giving the drug to children with dyslipidemia. Animals were treated with 0.9% saline, or rosuvastatin at 3 mg/day, and/or at 10 mg/day. Once the rats reached the age of puberty, they were euthanized, after which blood, the left testis, vas deferens, epididymis, and seminal gland were collected.

Testosterone, FSH, and LH were measured, and the histological structure of the testis was assessed. Following treatment, comparable measures of FSH and LH levels were observed; however, there was a decrease in testosterone. The two groups treated with rosuvastatin also displayed an increased tendency to abnormal seminiferous tubules with increased acidophilic cells. The experimental groups also experienced a delayed onset of puberty which may have been due to the decreased cholesterol [13]. These findings are supported by another study that found that prepubertal rats exposed to rosuvastatin were at an increased risk of decreased testosterone levels and sperm quality, both hindering the onset of puberty and affecting fertility [69]. A more recent study by Silva et al. confirmed the previous findings of Leite et al., by examining the effects of rosuvastatin in a cohort of Wistar rats. They found that rosuvastatin altered epididymal morphology, caused a delay in ejaculation, and as well a reduced fertility potential after natural mating [42].

In contrast, a study by Heeba et al. examined the anti-oxidative effects of rosuvastatin on diabetes-induced testicular damage in a cohort of male Wistar rats (180–210 g). After eight weeks of treating diabetic rats with either 5 mg/day or 10 mg/day of rosuvastatin, the animals were killed by cervical decapacitation, after which the testis and the epididymis were collected. The evaluation showed that diabetic rats displayed testicular degeneration and necrotic seminiferous tubules. The rosuvastatin treated groups maintained the weight of the epididymis and prevented severe degeneration of the seminiferous epithelium. Rosuvastatin reduced the levels of caspase 3, NF-κβ, and iNOS induced by high glucose levels, possibly owed to the anti-oxidative properties of rosuvastatin [43].

As seen in the previously discussed forms of statin, the contradictory outcomes in these animals’ studies are plausibly due to variability in disease conditions. The studies that reported the adverse effects of rosuvastatin were performed on healthy rats while the studies that showed beneficial effects were carried out on diseased animals. Taken together, rosuvastatin appears to have a detrimental effect on fertility in animal models, as healthy animals treated with rosuvastatin displayed a decrease in testosterone production and an altered spermatozoa quality. Additionally, rosuvastatin delayed the onset of puberty in prepubertal animals.

#### 6.4.2. Human Findings

A case report by Tada et al. showed that rosuvastatin was able to cause reversible azoospermia in a patient seeking fertility treatment. After 16 weeks of withdrawing from rosuvastatin intake, sperm was seen in the semen, making IVF possible [11]. Although clinical trials regarding the effect of rosuvastatin on male fertility are limited, these outcomes indicate that rosuvastatin adversely affected male reproductive parameters during active usage.

### 6.5. Simvastatin and Male Infertility

Simvastatin is a relatively short-lived statin that is taken at doses of 5–80 mg. In vitro studies have shown that simvastatin affects 17-hydroxy-steroid-dehydrogenase. This enzyme catalyzes the conversion of androstenedione to testosterone which is essential for the production of sperm [53]. Even though in vivo reports of simvastatin on male fertility are scarce, some have attempted to draw significant conclusions between the two.

#### 6.5.1. Animal Findings

Shalaby et al. examined the effects of simvastatin on male fertility in a cohort of 35 male albino Swiss hypercholesterolemic rats. Each of the rats was given simvastatin (40 mg) for 65 consecutive days. At days 0, 15, 30, 45, and 60, male rats were paired with four females to stimulate mating. On days 0, 30, and 60, blood samples were collected to assess levels of testosterone and cholesterol. On day 66, the rats were sacrificed, the testis, seminal vesicle, and prostate gland were removed. The study revealed no changes in testosterone levels; however oral simvastatin significantly (*p* < 0.001) increased the total sperm count, sperm motility, and viability. Oral administration of simvastatin was also found to maintain the weight of the testis and maintain the integrity of the seminiferous tubules [39].

#### 6.5.2. Human Findings

In human studies, Pruvis et al. examined the short-term effects of simvastatin (40 mg) in a cohort of 19 men with familial hypercholesterolemia for eighteen weeks. The participants of the study were exposed to a placebo for four weeks, followed by simvastatin for fourteen weeks. Findings showed that treatment was not associated with any significant change in sperm quality, the seminal plasma concentrations of different sex gland products (prostate-specific acid phosphatase, polyamines, citrate, fructose, and α-glucosidase), or the serum concentrations of cortisol, testosterone, LH, FSH, or prolactin [49]. It was therefore suggested that a short-term reduction in circulating LDL-cholesterol has no evident effect on testicular function or sperm quality.

Azzarito et al. performed a similar study, however, they examined the long-term effects of simvastatin on gonadal hormones. They administered simvastatin at 10 mg for the first six weeks of the study, followed by 20 mg for a further six weeks, and finally 40 mg until the end of the study (12 months) in a cohort of 10 hypercholesterolemia patients. Blood samples were drawn at months 0, 1.5, 3, 4.5, 6, 9, and 12. They reported that the levels of free testosterone did not change, and concluded that long-term therapy with high-dose simvastatin does not interfere with either adrenocortical function or sex hormone production [50].

Another study investigated the effects of simvastatin in a cohort of 18 male patients with non-insulin-dependent diabetes. The participants of this study were exposed to a placebo for four weeks, followed by simvastatin at 10 mg for two months. After the first month of simvastatin therapy, no changes were observed in testosterone, dehydroepiandrosterone sulfate (DHEA-S), LH, and FSH, while a significant increase was noted in androstenedione. After two months of treatment, the latter was confirmed, finding a significant increase in androstenedione [51]. The authors of this study hypothesized that this might be due to the inhibitory effect of simvastatin on 17-hydroxy-steroid dehydrogenase, which was observed by Smals et al. in 1991 [53].

Recently, a study by Al-Hilli et al. evaluated whether treatment with simvastatin (20 mg) for 12 weeks could improve the degree of lipid peroxidation, thus improving sperm formation and maturation. This study was conducted in a cohort of 90 patients (55 infertile and 35 control). The authors observed that simvastatin decreased the extent of lipid peroxidation in seminal plasma and spermatozoa, which was noted by an overall decrease in MDA levels. The reduced levels of oxidative stress indicated that simvastatin promoted sperm motility and viability, thus, serving as a beneficial treatment for male infertility [52]. On a very superficial level, when assessing the effects of statins on male fertility simvastatin seems to have the most beneficial effect when compared to the previous statins that were discussed. However, the studies presented are not conclusive. Larger randomized control trials are needed to accurately demonstrate the short and long-term effects of Simvastatin on male fertility.

### 6.6. Lovastatin and Male Infertility

Although studies that have evaluated the effect of lovastatin on overall male reproductive health are limited, the available findings show that lovastatin reduces steroidogenesis [18,70,71]. For instance, Tobert et al., reported that after administering lovastatin in multiple doses to healthy male volunteers for four weeks, the serum concentration of testosterone and other steroidal hormones was reduced [70]. The same trend of results was also reported by Mcdonald et al., who administered lovastatin in different doses to animals in a preclinical setting [71]. Although more recent studies evaluating these effects are lacking, it is unbiased to say that lovastatin could hamper spermatogenesis if the level of testosterone is drastically and sustainably reduced.

## 7. Evidence of the Effect of Statin on Male Sexual Health

**Figure 2:**
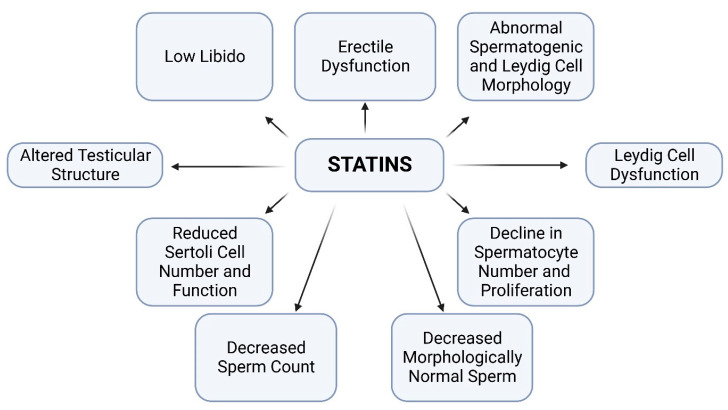
The summarized effects of statins on male sexual health and reproductive parameters. It is important to note that not all studies on the different forms of statins reported these adverse effects.

Findings on the effects of statins on male sexual health are currently controversial. Hall et al. in their earlier study reported that after measuring serum total testosterone, free testosterone, dehydroepiandrosterone sulfate, and LH in 1812 men on statins treatment, there was no association observed between the use of statins and the subsequent occurrence of erectile dysfunction [6]. The same group of authors in a different study showed that lipid-lowering medications may be associated with erectile dysfunction among some men [72]. Nevertheless, many other studies showed the negative impact of statins on male sexual health. The study of Bruckert et al. showed that men treated with statins complained more frequently of erectile dysfunction, with further multivariate analyses showing that impotence was dependent on treatment with statins [73]. Another study reported that eight patients placed on different statins such as simvastatin, fluvastatin, pravastatin, and atorvastatin showed decreased libido during treatment. Of these, two cases were further analyzed to determine the status of the levels of sex hormones. Results showed that testosterone was markedly decreased in these patients [74]. Testosterone has long been associated with sexual behavior as it enhances several steps of the entire male sexual response [75]. For instance, the study of Rastrelli et al. showed that reduced testosterone levels were positively correlated with severe sexual dysfunction symptoms such as erectile dysfunction, impaired morning erections, and low sexual desire (libido). Where both the total and the free testosterone were significantly lower in men with a low libido and severely reduced morning erections and a trend towards significance was observed for severe erectile dysfunction [76]. Additionally, the study of Smals et al. showed that treatment with simvastatin suppressed testicular hormone synthesis by selectively inhibiting the 17-ketosteroid-oxidoreductase enzyme activity at the latter stage of steroidogenesis [53]. These enzymes catalyze the conversion of dehydroepiandrosterone and androstenedione to androstenediol and testosterone, respectively. The summarized negative consequences of statins on male sexual health and reproductive parameters are described in Figure 2.

## 8. Mechanisms through which Statins May Impact Male Reproduction

Statins may adversely affect male sexual health and reproductive parameters by interfering with hormonal balance and normal testicular function.

For steroidogenesis to occur, the Leydig cells can absorb cholesterol from the blood via low density lipoprotein-receptors and are also capable of de novo cholesterol synthesis. Upon the initiation of statin metabolism, there is a decrease in the circulating LDL-cholesterol and the total amount of cholesterol transported to the Leydig cells is reduced [77]. However, the reduction in the amount of cholesterol transported to the Leydig cells does not necessarily infer impaired steroidogenesis. In lieu of this, the findings of studies that have shown the reduced expression and malfunctioning of LDL-receptors in patients with familial hypercholesterolemia appear important [74,78]. With these findings, it means that even if enough cholesterol is available for uptake by the Leydig cells after statin use, the incapability of the LDL-receptor to aid the transport into the Leydig cells has tampered with the bioavailability of cholesterol in these cells, thus making Ledig cells depend solely on the de novo synthesis of cholesterol. Additionally, since unretained statins are distributed to other organs including the testes, then, it is possible that the de novo cholesterol synthesis is also inhibited. Hence, there will be little or no bioavailability of cholesterol for testosterone synthesis [75]. Subsequently, the lack of or decrease in testosterone will lead to a reduced libido or in some cases, erectile dysfunction.

A lowered testosterone level is not only associated with altered male sexual health, but it is also connected to altered testicular function. Azzarito et al. reported a significant decrease in both the basal and human chorionic gonadotropin-stimulated free testosterone levels in patients receiving simvastatin after a prolonged treatment period (6–12 months), but the levels remained within a clinical range [8]. Several other studies have linked impaired hormonal balance to disrupted spermatogenesis and altered testicular and spermatogenic structures [60,79]. Although very debatable, findings from both animal and human studies have also proposed that a reduction in circulating testosterone caused an increase in the generation of pro-inflammatory cytokines, which is reviewed in detail by Mohamad et al. [80].

**Figure 3:**
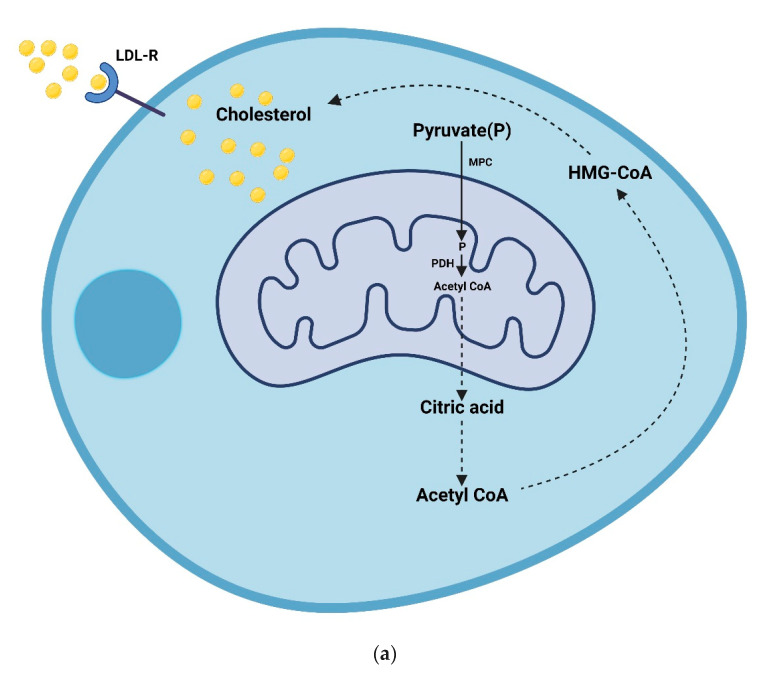

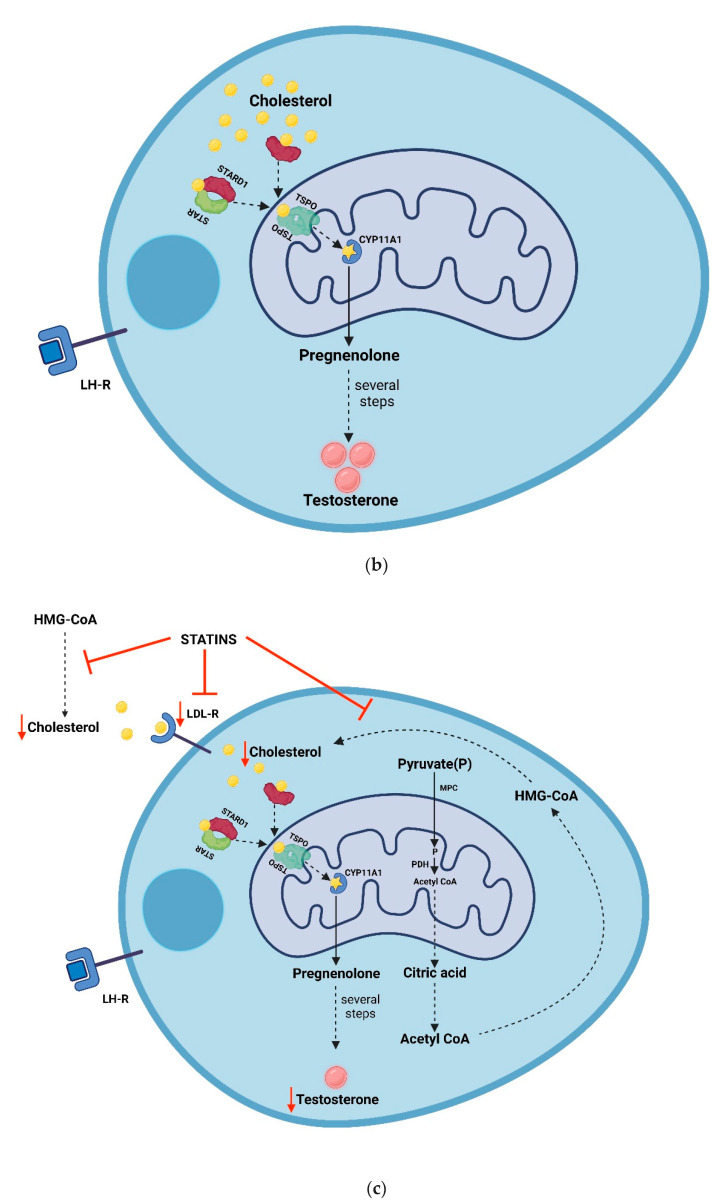
The mechanisms through which statin may impact male fertility. (**a**). Cholesterol bioavailability in a Leydig cell; (**b**). Testosterone synthesis in a Leydig cell; (**c**) Routes through which statins affect male reproduction. The Leydig cell can absorb cholesterol from the blood via low density lipoprotein- receptors (LDL-R) and are also capable of de novo cholesterol synthesis. The de novo synthesis of cholesterol starts with pyruvate being generated from diverse cytosolic sources. Entry of pyruvate into the mitochondrial matrix is mediated by the mitochondrial pyruvate carrier (MPC). In the matrix, pyruvate is converted into acetyl CoA or oxaloacetate by pyruvate dehydrogenase (PDH). The acetyl CoA produced is converted to citric acid outside the matrix. Upon the initiation of statin metabolism, there is a decrease in the circulating LDL-cholesterol and the total amount of cholesterol transported to the Leydig cell is reduced. Additionally, the incapability of the LDL-R to aid the transport into the Leydig cell will further tamper with the bioavailability of cholesterol in these cells, thus making the Ledig cell solely dependent on the de novo synthesis of cholesterol. Since the unretained statins are redistributed to other organs including the testes, then, it is possible that the de novo cholesterol synthesis is also inhibited. Hence, there will be little or no bioavailability of cholesterol for testosterone synthesis. Subsequently, the lack of or decrease in testosterone will lead to male sexual dysfunctions and altered reproductive parameters. TSPO (mitochondrial translocator protein 18); STAR (cytosolic steroidogenic acute regulatory protein); STARD1 (START domain-containing 1); ACBD3 (acyl-coenzyme A binding domain-containing 3); CYP11A1 (cytochrome p450 family 11 subfamily A polypeptide 1); MPC (mitochondrial pyruvate carrier); LH-R (luteinizing hormone receptor); PDH (pyruvate dehydrogenase); and HMG-CoA (3 hydro 3 methyl glutaryl co enzyme A).

Briefly, in an animal model of hypogonadism, where there are reduced testosterone levels, inflammatory cytokines such as IL-6, IL-1β, and TNF-α were said to be elevated and only IL-6 was reduced after testosterone treatment [81,82]. Similarly, findings from human studies have also shown that there are increased inflammatory cytokines in hypogonadism [83,84]. However, some other findings negate this outcome [85]. The contradictory findings may be due to differences in disease severity. Studies that reported the negative effects had investigated men with hypogonadism, while those that reported no association had enrolled men with mild hypogonadism or healthy men with partial androgen deficiency. All outcomes considered, a reduction in circulating testosterone can have a negative impact on overall male fertility. Figure 3 shows the summary of the possible route through which statins could affect testosterone production and thereafter cause sexual dysfunction, altered reproductive parameters, and aid increased pro-inflammatory cytokines.

## 9. Addressing the Problem: Should Antioxidants Be Administered along with Statins?

The evaluation of the current literature reveals that the use of the different forms of statins and the subsequent effect on male fertility remains inconclusive and have left room for further discussion on how to address the differences in results and how to mitigate any adverse effects that might arise as a consequence of inhibiting HMG-CoA reductase.

Studies have shown the deleterious effects of high levels of cholesterol and LDL on male fertility [86,87], and statins have been proven [88] to be an efficient drug of choice to treat elevated levels of LDL and cholesterol from various etiologies such as a high fat diet, obesity, and familial hypercholesterolemia. Other benefits of statins stem from their pleiotropic effects on the mevalonic pathway, and their anti-inflammatory, immunomodulatory, and anti-thrombotic properties.

However, on the flipside, statins have several reported side effects including, but not limited to myopathy [89], diabetes [90], and hemorrhagic strokes [91]. These complications arise in part due to the development of oxidative stress, which can be alleviated with the use of antioxidants.

There have been studies that have investigated the combination therapy of antioxidants with statins to suppress the statins’ oxidative stress. The results have been promising, with a study concluding that a grape seed extract and atorvastatin combination treatment showed a noticeable increase in serum testosterone and sperm count whilst reducing levels of superoxide dismutase, caspase, and glutathione. Some studies have also shown the enhanced effects of antioxidants given alongside a statin with the antioxidants’ ability to diminish the statins’ adverse effects [92]. Conversely, one study showed the dose-dependent antioxidant and anti-apoptotic properties of rosuvastatin on diabetes-induced reproductive damage [43]. In the case of the latter report, rosuvastatin was shown to exert the antioxidant effect, which opens an avenue to initiate a different conversation.

## 10. Future Recommendation

From the available evidence, although inconclusive, the findings have shown that some forms of statins can cause testicular dysfunction owing to their ability to reduce testosterone production. Therefore, we recommend that future studies should investigate how these adverse effects are exerted, aside from the causation of reduced steroid hormones.

A cue into the investigation is that during the hepatic uptake of statins, the influx is aided by the organic anion transporter polypeptides (OATP) and is encoded by the solute carrier superfamily genes. In the instance that statins are not entirely taken up by the liver cells, the remaining statins are distributed to other organs of the body through systemic circulation. Future investigation should now include whether the testis, the blood-testis-barrier, the Sertoli cells, and even the Leydig cells and other spermatogenic cells have these transporters and whether their function is the same in these cells. Additionally, testicular pain/discomfort is reported to be part of the statin-associated effects. Hence, studies should focus on the pathways through which this could occur.

## 11. Conclusions

Statins undoubtedly remain the gold standard therapy for hypercholesterolemia. However, many side effects are still being reported. Although the influence of statins on fertility is not widely investigated, multiple studies have demonstrated their effects on fertility. Both animal and human studies present conflicting data on whether statins do, in fact, influence fertility outcomes. However, it is evident that reduced testosterone may lead to a variety of male reproductive impairments. These contradictions are in part due to the different age of users, the concentrations of statins, the type and duration of treatment, and the underlying disease and/or comorbidity. Nevertheless, it is still important to address this discrepancy in the research studies as it is a common medicine in use and usually the medication is initiated at a young age and continued throughout life. Therefore, larger experimental and clinical studies are needed mainly on humans to draw any definitive conclusions on the effects of statins on male fertility.

## Figures and Tables

**Table 1:** Summary of studies that evaluated the effect of statins on male reproductive parameters.

Ref/Author	Country	Year	Study Population	Type of Statin	Statin Dose	Effect of Statin on Fertility
**Animal findings**						
[36] Akdeniz et al.	Turkey	2020	Animal	Atorvastatin	20 mg	(−) Decrease in the number of Sertoli cells, Spermatogonia, Spermatocytes. (−) Decrease in seminiferous tubule diameter. (−) Loss of cellular boundaries in the testes. Overall effected spermatogenesis
[37] Zangoie et al.	Iran	2019	Animal	Atorvastatin	100 mg/kg	(+) Increased the amount of viable sperm. (+) Prevented testicular injury against busulfan.
[38] Naeimi et al.	Iran	2017	Animal	Atorvastatin	10/20/50 mg/kg	(−) Decreased radiation induced oxidative stress. (−) Decreased level of testosterone. (+) Increased testicular tubal diameter and epithelial thickness
[39] Shalaby et al.	Egypt	2003	Animal	Simvastatin	40 mg daily	(+) Increased the weight of the testes. (+) Increased sperm count. (+) Increased sperm motility. (−) Decreased sperm abnormalities. (+) Maintained the structure of the seminiferous tubules.
[40] Cui et al.	China	2017	Animal	Fluvastatin	6 mg/kg	(+) Reversed spermatogenic damage. (+) Protective effect on the seminiferous tubules. (+) Protective against testicular atrophy. (+) Protective effect on spermatogonia, Sertoli cells, and Leydig cells. (+) Increases the expression of mTOR.
[41] Gurel et al.	Turkey	2019	Animal	Fluvastatin	6 mg/kg	(−) Reduced oxidative stress. (+) Increased testosterone levels. (+) Protective effect on seminiferous tubules and spermatogenic cells. (+) Protective effect on sperm count.
[13] Leite et al.	Brazil	2014	Animal	Rosuvastatin	3 or 10 mg/kg	(−) Decrease testosterone concentration. (−) Delayed epididymal development. (+) Promoted the development of abnormal seminiferous tubules.
[14] Leite et al.	Brazil	2017	Animal	Rosuvastatin	5 and 40 mg daily	(−) Testosterone was decreased. (−) Decreased fertility, based on post-implantation loss. (−) Decreased production of sperm and mature spermatids. (−) Reduction in the rate of progressive sperm. (+) Increased sperm head abnormalities. (−) Loss of structure of the seminiferous tubules.
[42] Silva et al.	Brazil	2020	Animal	Rosuvastatin	5 mg/kg	(−) Reduced ejaculation frequency. (−) Hyperplasia of clear cells in the proximal region of the cauda epididymis.
[43] Heeba et al.	Egypt	2015	Animal	Rosuvastatin	10 mg/kg	(+) Improved sperm count and motility. (−) Decreased testicular nitric oxide. (+) Maintained the weight of the testes and structure of the epididymis. (−) Showed less degeneration of seminiferous tubules with shedding of germ cells.
[44] Farsani et al.	Iran	2018	Animal	Pravastatin	20 mg/kg	(+) Maintained testicular volume, seminiferous tubule diameter, and germinal epithelium. (+) Maintained epididymal sperm count. (+) Maintained the number of spermatogonia, spermatocytes, and Sertoli cells. (+) Showed radical scavenging properties.
[45] Dursun et al.	Turkey	2014	Animal	Pravastatin	30 mg/kg	(+) Showed antioxidant properties.
[44] Esrafil et al.	Iran	2017	Animal	Pravastatin	20 mg/kg	(−) Reduced doxorubicin induced oxidative damage. (+) Showed antioxidative effects.
[46] Kassan et al.	Spain	2010	Animal	Pravastatin		(+) Showed antioxidant properties. (−) Reduced lucigenin induced O_2_ levels.
**Human findings**						
[47] Pons-Rejraji et al.	France	2014	Human	Atorvastatin	10 mg	(−) Decreased the number of spermatozoa. (−) Decreased vitality, motility, and morphology of sperm. (−) Alterations in prostatic and epididymal function. (−) Decreased spontaneous acrosome reacted spermatozoa.
[19] Bernini et al.	Italy	1998	Human	Pravastatin	20 mg/day	(+/−) No change in testosterone. (+/−) No change in motility, morphology, and sperm count.
[48] Travia et al.	Italy	1995	Human	Pravastatin	40 mg/day	(+/−) No change in testicular steroidogenesis. (+/−) No change adrenocortical function.
[9] Dobs et al.	U.S.A	1993	Human	Pravastatin	20 to 80 mg daily	(+/−) No change in testosterone levels. (−) Decreased sperm motility.
[11] Tada et al.	Japan	2015	Human	Rosuvastatin	2.5 mg daily	(−) Decreased spermatozoa. (−) Decreased sperm count. (−) Decreased sperm motility.
[49] Purvis et al.	Norway	1992	Human	Simvastatin	40 mg daily	(+/−) No effect on the quality of sperm. (+/−) No effect on testosterone.
[50] Azzarito et al.	Italy	1992	Human	Simvastatin	20–40 mg daily	(+/-) No change in testosterone.
[51] Rossato et al.	Italy	1993	Human	Simvastatin	10 mg daily	(+) Increased levels of androstenedione. (−) Thought to have an inhibitory effect on 17-hydroxysteroid-de-hydrogenase.
[52] Al-Hilli et al.	Iraq	2017	Humans	Simvastatin	20 mg daily	(+) Improved sperm motility. (−) Reduced the degree of lipid peroxidation. (+) Improved sperm morphology and viability. (+) Improved sperm formation and maturation.
**Mammalian tissue/cells**						
[53] Smals et al.	The Netherlands	1991	Human Testicular Tissue	Simvastatin	0.01 and 0.1 μM	(+) A dose of more than 0.1 μM Effects 17-hydroxysteroid-de-hydrogenase.
[54] Imaeda et al.	Japan	2001	Mammalian cells	Fluvastatin	1–10 mM	(−) Decreased DNA tail movement. (−) Reduced DNA damage. (+) Presented radical scavenging properties
[55] Okubo et al.	Japan	2019	Renal Cancer Cells	Fluvastatin		(+) Shows increased mTOR expression.
[56] Wang et al.	China	2021	Neuronal Cells	Fluvastatin		(+) Fluvastatin reduced O_2_ induced damage of neuronal cells in a dose dependent manner.

(−) = negative or adverse effect; (+) = Positive effect.

## Data Availability

Not applicable.
